# Laser-Induced Graphene Arrays-Based Three-Phase Interface Enzyme Electrode for Reliable Bioassays

**DOI:** 10.3390/biomimetics8010026

**Published:** 2023-01-08

**Authors:** Man Zhang, Jun Zhang, Zhenyao Ding, Haili Wang, Lihui Huang, Xinjian Feng

**Affiliations:** 1 College of Chemistry, Chemical Engineering and Materials Science, Soochow University, Suzhou 215000, China; 2 Innovation Center for Chemical Science, Soochow University, Suzhou 215000, China

**Keywords:** laser-induced graphene, three-phase interface, oxidase kinetics, electrochemical biosensor

## Abstract

Electrochemical oxidase biosensors have been widely applied in healthcare, environmental measurements and the biomedical field. However, the low and fluctuant oxygen levels in solution and the high anodic detection potentially restrict the assay accuracy. To address these problems, in this work, we constructed a three-phase interface enzyme electrode by sequentially immobilizing H_2_O_2_ electrocatalysts and an oxidase layer on a superhydrophobic laser-induced graphene (LIG) array substrate. The LIG-based enzyme electrode possesses a solid–liquid–air three-phase interface where constant and sufficient oxygen can be supplied from the air phase to the enzymatic reaction zone, which enhances and stabilizes the oxidase kinetics. We discovered that the enzymatic reaction rate is 21.2-fold improved over that of a solid–liquid diphase system where oxygen is supplied from the liquid phase, leading to a 60-times wider linear detection range. Moreover, the three-phase enzyme electrode can employ a cathodic measuring principle for oxidase catalytic product H_2_O_2_ detection, which could minimize interferences arising from oxidizable molecules in biofluids and increase the detection selectivity. This work provides a simple and promising approach to the design and construction of high-performance bioassay systems.

## 1. Introduction

Oxidase-based electrochemical biosensors have been extensively used and studied due to their high efficiency and favorable selectivity [1,2,3]. Typically, oxidase oxidizes the substrate (analyte) while consuming oxygen and producing H_2_O_2_ [4,5]. The analyte level is subsequently determined via the electrochemical detection of H_2_O_2_. The anodic measurement of H_2_O_2_ commonly requires high potentials, in which numerous endogenous/exogenous molecules in biofluids are oxidized [6,7]. Obviously, sufficient oxygen supply and cathodic measurement of H_2_O_2_ at negative potentials are more conducive to high-performance biosensors [8]. However, the dissolved oxygen level at the solid/liquid reaction interface of the conventional diphase biosensor is relatively low and fluctuant, which directly affects oxidase kinetics and the formation rate of H_2_O_2_, in turn, limiting detection performance. Furthermore, the cathodic measurement of H_2_O_2_ is also restricted by fluctuant oxygen levels because oxygen can be reduced at similar potentials, in turn, reducing the detection accuracy.

Inspired by natural non-wetting surfaces [9,10], artificial superhydrophobic materials have been extensively fabricated and show great potential in many areas [11,12,13,14]. When superhydrophobic substrate is exposed to an aqueous solution, air pockets become trapped in the gaps of the solid/liquid interface, forming a gas–solid–liquid three-phase interface that allows oxygen to reach the solid-surface reaction zone from the gas phase (constant and sufficient) [15,16]. This provides an opportunity and basis for the fabrication of high-performance oxidase-based electrodes. Various superhydrophobic substrates have been reported to prepare three-phase oxidase electrodes [17,18,19,20], such as superhydrophobic carbon-based substrates, elastomer micropillar arrays, poly(vinylidene fluoride) membrane, and metal oxide nanowire arrays. These methods always have expensive and complicated manufacturing steps, which makes continuous fabrication difficult and limits practical applications. Therefore, there is an urgent need for simple and cost-effective approaches for the construction of reliable three-phase electrodes at scale.

**Figure 1:**
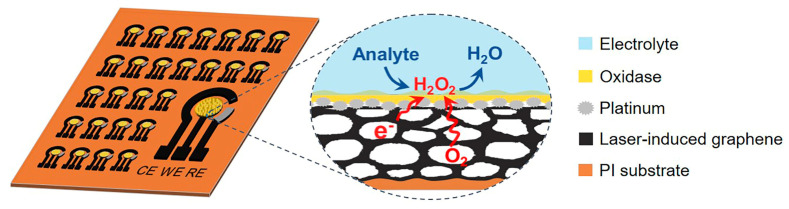
Schematic illustration of the fabrication of three-phase biosensor arrays (left). The working electrode (WE) of the three-phase enzyme electrode consists of a superhydrophobic LIG substrate, an H_2_O_2_ electrocatalyst layer, and a top oxidase enzyme layer. The right shows an enlarged view of the solid–liquid–air three-phase bio-electrocatalysis reaction process.

Recently, the CO_2_ laser-engraving technique, as a promising fabrication method, has received significant attention due to its various advantages, including cost-effectiveness, no mask and high yield. The three-dimensional (3D) porous laser-induced graphene (LIG) structure can be constructed by a one-step CO_2_ laser direct-writing method [21,22,23]. Due to its excellent conductivity, mechanical strength and scalable manufacture, LIG is considered as a prospective new candidate in electrochemical sensors [24,25,26]. This provides an ideal method for the scalable manufacturing and widespread implementation of three-phase biosensors. In this paper, we manufactured 3D porous conductive LIG substrate on polyimide (PI) film with CO_2_ laser-engraving technology. Based on the LIG substrate, a three-phase enzyme electrode with high performance was constructed in batches, as shown in Figure 1. The three-phase enzyme electrode consists of a H_2_O_2_ electrocatalyst-modified superhydrophobic LIG substrate and a top oxidase layer. During the detection process, analyte solution cannot be immersed underneath the porous hydrophobic LIG substrate of the electrode, leading to a solid–liquid–gas interface [15,16]. Then, sufficient and constant oxygen can be directly supplied from the gas phase to the oxidase layer, which, in turn, enhances the performance of the three-phase biosensor, including the detection linear range and accuracy. Additionally, the cathodic measurements of oxidase catalytic product H_2_O_2_ are also utilized to minimize interferences, since the constant interfacial oxygen level can generate a stable oxygen reduction current. The laser direct-writing technique–as a simple, economical and efficient method—provides a new approach to simplify the preparation process of three-phase biosensors.

## 2. Materials and Methods

### 2.1. Chemicals

Polyimide (PI) films with a thickness of 125 μm (Kapton^®^) were purchased from Weishengte Technology Co., Ltd. (Beijing, China). Polydimethylsiloxane (Sycgard^TM^ 184 silicone) was acquired from Dow Corning (Midland City, MI, USA). Dihydrogen hexachloroplatinate hexahydrate (H_2_PtCl_6_·6H_2_O) was obtained from Energy Chemical (Shanghai, China). Glucose oxidase (GOx, EC 1.1.3.4, 123 kU/g) was purchased from Toyobo Co., Ltd. (Shanghai, China). Bovine Serum Albumin (BSA), lactate oxidase, sucrose invertase and ethanol oxidase were acquired from Yuanye Bio-Technology Co., Ltd. (Shanghai, China). Hydrogen peroxide was purchased from Shanghai Lingfeng Chemical Reagent Co., Ltd. Chitosan and glutaraldehyde (25%) were obtained from Aladdin. Cyclohexane, potassium chloride, disodium hydrogen phosphate, sodium dihydrogen phosphate, glucose, sucrose, ethanol, lactate, methanol, mannose, xylose, galactose, citric acid and CH_3_COOH were purchased from Sinopharm Chemical Reagent Co., Ltd. (Shanghai, China). All of our experiments used deionized (DI) water. All reagents were directly used without further purification.

### 2.2. Fabrication of LIG-Based Three-Phase Biosensor

#### 2.2.1. Preparation of Laser-Induced Graphene Substrate (LIG)

The PI film (thickness of 125 μm) was wiped with ethanol before use. After drying in the air, it was attached to an aluminum alloy plate for laser scribing. Then, a pre-designed three-electrode pattern (the detection area is a circle with a diameter of 4 mm) was imported into a CO_2_ laser system. Subsequently, the cleaned PI film was subjected to laser irradiation by the CO_2_ IR laser, with a wavelength of 10.6 μm, to generate a porous LIG substrate. The LIG was prepared using a power of 6.0 W at a scan rate of 70 mm/s.

#### 2.2.2. Hydrophobic Treatment of LIG Substrate

The as-prepared LIG substrate was immersed in a 30-fold cyclohexane-diluted polydimethylsiloxane (PDMS) solution for 30 min, removed and cured at 120 °C for 30 min.

#### 2.2.3. Electrodeposition of Pt Nanoparticles and Modification of the Enzyme Layer

The mixture H_2_PtCl_6_ solution (10 g/L H_2_PtCl_6_: H_2_O: 1 M H_2_SO_4_ = 1:1:2, v:v:v) was used for the electrodeposition of Pt NPs. Before deposition, the superhydrophobic LIG substrate was sputter-coated with gold via the sputtering (E-1010, Hitachi) of a gold target (99.99%) at power of 30 W and working pressure of 10 Pa, for 20 s, under an Ar atmosphere. Then, the Pt electrocatalysts were electrodeposited onto the Au-modified LIG substrate at −0.3 V versus Ag/AgCl for 10 s in the H_2_PtCl_6_ solution. Next, the 5 μL of a mixed solution of glucose oxidase (GOx, 20 mg mL^−1^ in DI water), chitosan (2 mg mL^−1^, in 1 vol% CH_3_COOH), glutaraldehyde (5 wt% in DI water) and DI water with a volume ratio of 20:10:1:9 was drop-cast onto the Pt/LIG substrate and dried under ambient conditions, thus, forming a three-phase glucose oxidase electrode. Other oxidase-based biosensors are similar to the above methods; the only requirement is to change the mixed oxidase solution. For the detection of lactic acid, 10 µL of a mixed solution of lactic acid oxidase (10 mg mL^−1^ in DI water), BSA (10 mg mL^−1^ in DI water) and glutaraldehyde (0.25 wt% in DI water), with a volume ratio of 5:5:3, was used. For the detection of sucrose, 5 µL of a mixed solution of sucrose invertase (40 mg mL^−1^ in DI water), glucose oxidase (20 mg mL^−1^ in DI water), chitosan (2 mg mL^−1^, in 1 vol% CH_3_COOH) and glutaraldehyde (5 wt% in DI water), with a volume ratio of 20:20:10:3, was used. For the detection of ethanol, 10 µL of a mixed solution of ethanol oxidase (1196 U mL^−1^), BSA (15 mg mL^−1^ in DI water), glutaraldehyde (5 wt% in DI water) and DI water, with a volume ratio of 10:20:1:20, was used. For the controlled experiment, the LIG-based diphase electrodes are also similar to the above methods, except that there is no PDMS treatment.

#### 2.2.4. Preparation of Reference Electrodes and Counter Electrodes

After finishing the preparation of the middle working electrode (three-phase enzyme electrode) and covering it with a mask, the LIG-based superhydrophobic three-electrode substrate was treated by long-time (5 min) oxygen plasma to produce the hydrophilic counter electrode (LIG). Finally, about 2 mg of Ag/AgCl ink was coated on the position of the reference electrode (~0.04 cm^2^) and baked at 60 °C for 15 min to produce the reference electrode (LIG/Ag/AgCl).

### 2.3. Characterization and Electrochemical Measurements

Three-dimensional porous LIG was prepared on the PI films using a laser-engraving machine equipped with a CO_2_ laser of 10.6 μm (SCE4030, Wuhan Sunic Photoelectricity Equipment Manufacture Co., Ltd., Wuhan, China). Morphologies were characterized by using FE-SEM (SU8010, Hitachi, Tokyo, Japan), and the same instrument was also used to acquire EDS to determine the distribution of Pt on the surface of the LIG substrate. The chemical composition of the LIG was investigated by a microscopic confocal Raman spectrometer (inVia reflex) with a 532 nm excitation laser. A CHI-660E work station (CH Instruments, Inc., Shanghai, China) was employed for all electrochemical measurements at room temperature. The water contact angle (CA) was measured using a CA goniometer (JC2000D6, Powereach, Shanghai, China).

### 2.4. Electrochemical Testing

Electrochemical characterization was performed at room temperature using a CHI 660E electrochemical workstation with a three-electrode system. The as-prepared three-phase electrode (WE, Figure 1) was used as the working electrode; the LIG was used as the counter electrode (CE, Figure 1); the homemade LIG/Ag/AgCl (RE, Figure 1) was used as the reference electrode. The 0.2 M phosphate buffer solution (PBS, pH 7.2) was used as the electrolyte. Amperometric experiments were all performed at 0 V vs. Ag/AgCl with a stirring rate of 600 rpm during measurements. In different oxygen concentration experiments, the PBS buffered solutions were deoxygenated by bubbling with nitrogen. While stirring the deoxygenated solution, the oxygen can diffuse from air to the liquid phase. A dissolved oxygen meter (MP516, SANXIN, China) was employed to record the oxygen concentrations of the solution. In selectivity testing, after the addition of 1 mM of glucose using the LIG-based three-phase biosensor, a series of interferents (0.1 mM of methanol, ethanol, sucrose, lactic acid, galactose, xylose, mannose and citric acid) were added to the solution.

## 3. Results and Discussion

**Figure 2:**
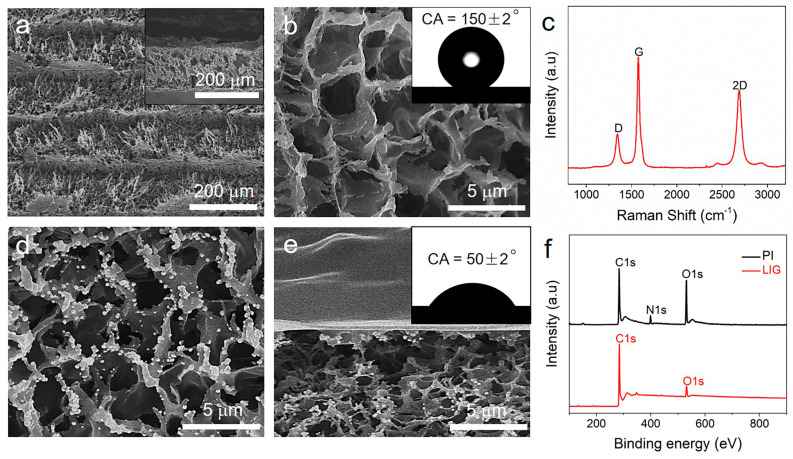
Characterization of the obtained LIG-based three-phase enzyme electrode. (**a**) Scanning electron microscopy (SEM) top view of the LIG substrate. The inset shows a SEM side view of the LIG substrate. (**b**) SEM image of the LIG substrate with polydimethylsiloxane (PDMS) treatment; the inset shows a water droplet placed on the substrate with a CA of about 150 ± 2º. (**c**) Raman spectrum of a LIG. (**d**) SEM image of the Pt electrocatalysts deposited on the LIG porous substrate. (**e**) SEM image (side view) of LIG-based three-phase enzyme electrode; the inset shows a water droplet placed on the enzyme electrode with a CA of about 50 ± 2º. (**f**) XPS characterization of PI and LIG.

Based on the three-phase biosensor model presented above, a CO_2_ laser-engraving machine was used to fabricate the superhydrophobic enzyme electrode substrate (see details in Materials and Methods). In this work, glucose oxidase was selected as a model enzyme, and the fabrication process of the biosensor is shown in Figure S1. Typically, a commercial PI film was first directly converted to the LIG structure by CO_2_ laser engraving. The overall characteristic morphology of the obtained LIG is shown in Figure 2a. Due to the rapid release of gaseous products under extremely high localized temperature, the obtained LIG shows a 3D porous interconnected structure, which has a wide pore size range of 1–10 μm (Figure 2b) and a thickness of about 140 μm (Inset of Figure 2a). Figure 2c shows the Raman spectrum of the obtained LIG, which contains three characteristic peaks: the D peak at 1346 cm^−1^, the G peak at 1577 cm^−1^ and the 2D peak at 2692 cm^−1^. The strength and symmetry of the 2D band is the fingerprint signal of graphene. The D/G intensity ratio (0.31) indicates the formation of the porous 3D graphene foam with relatively high quality [27,28]. As shown in Figure 2f, X-ray photoelectron spectroscopy (XPS) showed that the N/O ratios were sharply suppressed and the C/O ratios were enhanced after converting PI to LIG, indicating a high degree of carbonization [29]. Then, in order to strengthen the toughness of the LIG substrate and improve its surface hydrophobic properties, the as-produced LIG substrate was immersed in PDMS solution diluted with cyclohexane, taken out, dried at room temperature and cured at 120 °C for 1 h. The water contact angle (CA) of the LIG substrate after PDMS treatment was about 150 ± 2º (inset of Figure 2b), indicating super-hydrophobicity. Next, H_2_O_2_ electrocatalyst Pt particles with an average diameter of 250 ± 50 nm were electrodeposited on the LIG substrate (Figure 2d). The existence and distribution of the Pt particles were confirmed by elemental mapping from Figure S2. Finally, a mixed glucose oxidase/chitosan solution was drop-casted onto the Pt-loaded LIG surface with a fixed area to form the enzyme electrode. Figure 2e shows that the thickness of the oxidase-chitosan composite layer was about 1 µm. The insert in Figure 2e shows a water droplet placed on the surface of the enzyme electrode with a water CA of 50 ± 2º, indicating the formation of a hydrophilic surface. In the three-phase enzyme electrode, oxygen and electrons can transport to the enzyme reaction region through the gas phase and the cross-linked graphene, respectively. For comparison, a diphase oxidase electrode was fabricated by using a LIG without hydrophobic treatment. The detailed fabrication process of the diphase electrode is shown in the Materials and Methods.

**Figure 3:**
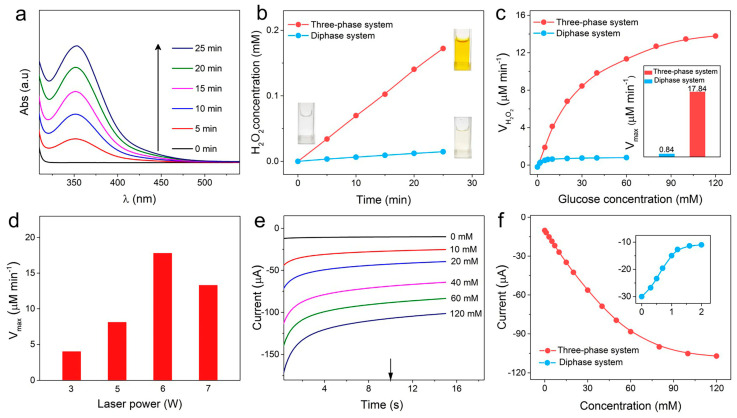
(**a**) UV-vis absorption (Abs.) spectra of oxidase enzymatic product H_2_O_2_ versus reaction times at 20 mM glucose level based on the three-phase system. (**b**) The linear relation of H_2_O_2_ concentration with reaction time in three-phase and diphase systems. The inset photos show the color change with the enzyme-catalyzed reaction time in iodometry on three-phase and diphase electrodes. (**c**) The functional relation of H_2_O_2_ production rate with glucose concentration based on three-phase and diphase systems. The inset shows the V_max_ using diphase (blue column) and three-phase (red column) systems. (**d**) The dependence of the V_max_ of the LIG-based three-phase systems on laser power. (**e**) Amperometric i-t curves of the LIG-based three-phase biosensors in glucose solution with concentrations from 0 to 120 mM in PBS solution at 0 V versus (vs.) Ag/AgCl. (**f**) Corresponding calibration plot derived from Figure 3e and Figure S7 at 10 s for the LIG-based three-phase and diphase biosensors.

To evaluate the oxygen permeability to the oxidase reaction zone, the enzyme reaction kinetics of a LIG-based three-phase sensor were first evaluated. Using iodometry, H_2_O_2_ concentrations can be calculated from the UV-vis absorption spectrum by the Beer-Lambert law (Equation (1)) (Figure S4). Figure 3a and Figure S3 show the absorbance peaks of the enzymatic product H_2_O_2_ over time after the addition of 20 mM of glucose into three-phase or diphase systems. The three-phase electrode exhibits a higher H_2_O_2_ generation rate and a more prominent color change of chromogenic solution compared with the diphase electrode, as shown in Figure 3b. The result demonstrates that the introduction of LIG-based three-phase substrate effectively improves the catalytic performance of the oxidase. To further investigate the kinetic performance of the enzyme, the chromogenic changes after 5 min upon the addition of glucose with different concentrations and corresponding initial H_2_O_2_ formation rates (V_0_) are plotted in Figure 3c and Figure S5. As the dose-dependent graphic is consistent with the Michaelis-Menten kinetic mechanism’s unique characteristic [30], the maximum velocity (V_max_) of the three-phase electrode can be calculated (Equation (2)) to be 17.84 μM min^−1^, a value of 21.2-fold higher than that of the diphase electrode (0.84 μM min^−1^), as shown in the insert of Figure 3c. The LIG-based three-phase electrode shows much-higher oxidase kinetics due to the high oxygen accessibility via the air phase to the reaction interface, which proves that the three-phase interface for the oxidase reaction was successfully constructed by using the LIG substrate.

(1) A=kbc

(2) $$V_{0}=\frac{V_{max}\cdot \left[S\right]}{K_{m}+\left[S\right]}$$

Laser power is the main influencing factor in the morphology and conductivity of the LIG [31]. We characterized the surface morphology and sheet resistance (R_s_) of LIG substrates with different laser preparation power. As the carbonization of PI has just begun, the surface morphology of the LIG was smooth without a 3D porous structure at a power of 3 W (Figure S6a). As the laser power increased, the PI film subjected to the photothermal effect generated gas and released rapidly, forming a 3D porous structure (Figure S6b,c). Once the power of the laser exceeded 6 W, the complete 3D porous geometry could not be maintained due to excessive thermal shock and structural destruction (Figure S6d). Meanwhile, the oxidase reaction kinetics based on the LIG substrate fabricated with a different laser power was assessed, as shown in Figure S6e. Since the number of three-phase contact point LIG offers increases as the 3D structure is more complete, V_max_ reached a maximum at 6 W, as shown in Figure 3d. Moreover, LIG electrodes exhibited a small sheet resistance with a minimum value of 7.26 Ohm at 6 W in virtue of the high conductivity of graphene (Figure S6f), which ensured excellent electron transfer as the substrate for the biosensor. Consequently, a laser power of 6 W was employed for the fabrication of the LIG-based electrode in the following test.

We then investigated the detection performance of the LIG-based three-phase electrode at 0 V (vs. Ag/AgCl). Amperometric i-t curves (Figure 3e) show that the electroreduction current continuously increased with the increase in glucose content up to the value of 120 mM. As shown in Figure 3f, the linear detection range of the three-phase biosensor reached 60 mM with a sensitivity of 12.34 μA·mM*^−^*^1^·cm*^−^*^2^. In sharp contrast, as the concentration of glucose rose, there was a decrease in the apparent cathodic current for the diphase biosensor, and a remarkably low upper detection limit was attained (~1.0 mM) (inset of Figure 3f and Figure S7). This is mainly because the increase in the cathodic current caused by the H_2_O_2_ of the oxidase reaction was counteracted by the decrease in the current resulting from oxygen consumption in the oxidase reaction. These results demonstrate that high-performance three-phase oxidase biosensors were successfully prepared by the LIG substrate.

**Figure 4:**
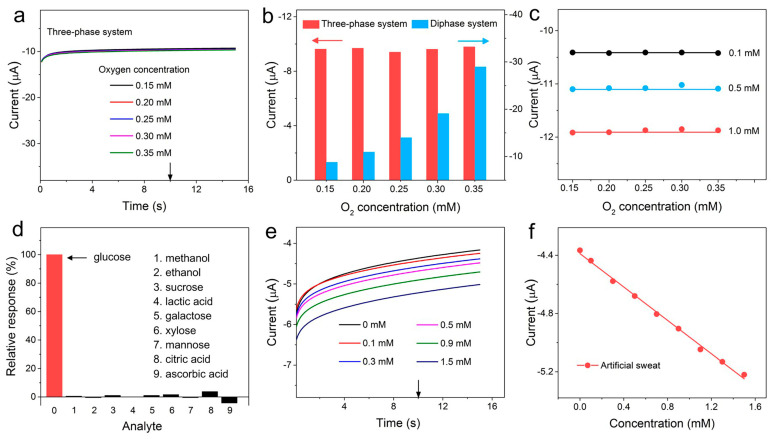
(**a**) Amperometric responses of LIG-based three-phase biosensors in blank PBS solution with different oxygen levels. (**b**) Background currents of the LIG-based three-phase and diphase biosensors in a PBS solution with various oxygen contents at 0 V versus (vs.) Ag/AgCl. (**c**) Amperometric response of the LIG-based three-phase biosensors when operated in 0.1, 0.5 and 1 mM glucose solutions with different oxygen contents. (**d**) Selectivity of the LIG-based three-phase biosensors with the addition of 1 mM of glucose and 0.1 mM of common interfering species. (**e**) Amperometric i-t responses of the LIG-based three-phase biosensors for glucose detection in artificial sweat. (**f**) Corresponding calibration plot derived from (**e**) at 10 s.

Since the cathodic current of oxygen overlaps that of H_2_O_2_ in the detection process, we investigated the effect of dissolved oxygen concentration on the detection current in LIG-based biosensors. The background currents were first studied with dissolved oxygen content variation on the three-phase and diphase biosensors. Before the experiment, the phosphate-buffered saline (PBS) solution was bubbled with nitrogen for 30 min to remove oxygen; then, the current response was recorded as oxygen and was introduced into the solution by stirring. As shown in Figure 4a,b (red columns), for the three-phase electrode system, negligible current fluctuations were observed as the dissolved oxygen level varied, suggesting that the oxygen level at the surface of the electrode was constant by virtue of a stable oxygen supply from the air phase. Due to the sufficient and stable oxygen level, both the oxygen reduction current (background current) and oxidase kinetics became stable in the three-phase biosensor. Consequently, the response current of the three-phase biosensor to 0.1, 0.5 and 1 mM of glucose was also constant without being affected by the dissolved oxygen level, as shown in Figure 4c and Figure S9a. For a diphase biosensor, as shown in Figure 4b (blue columns) and S8, the cathodic current increases with dissolved oxygen concentration because interfacial oxygen only comes from the liquid phase. From Figure S9b, it can be seen that the 1 mM glucose response current gradually increased along with the dissolved oxygen concentration. These results demonstrate that due to the stable interfacial oxygen level and oxidase kinetics, the current output of the three-phase enzyme electrode towards a fixed glucose concentration is insensitive to the dissolved oxygen content variety. This provides an opportunity to employ a cathodic measurement of H_2_O_2_ to eliminate interferences from many oxidizable species in biofluids and enhance detection selectivity. With reference to Figure 4d and Figure S10, the addition of 0.1 mM of methanol, ethanol, sucrose, lactic acid, galactose, xylose, mannose, citric acid and ascorbic acid into 1 mM of glucose resulted in a negligible variation in current, indicating the high selectivity of the LIG-based three-phase biosensor. Furthermore, as shown in Figure S11, the three-phase electrode also exhibited good detection abilities in the region of low concentration (0~1 mM), with a detection limit (LOD) as low as 0.05 mM. Then, the LIG-based three-phase biosensor was applied to detect glucose in artificial sweat. Figure 4e shows that the response current steadily increased with successive additions of glucose, and a good linearity in the range of 0 to 1.5 mM is observed (Figure 4f).

**Figure 5:**
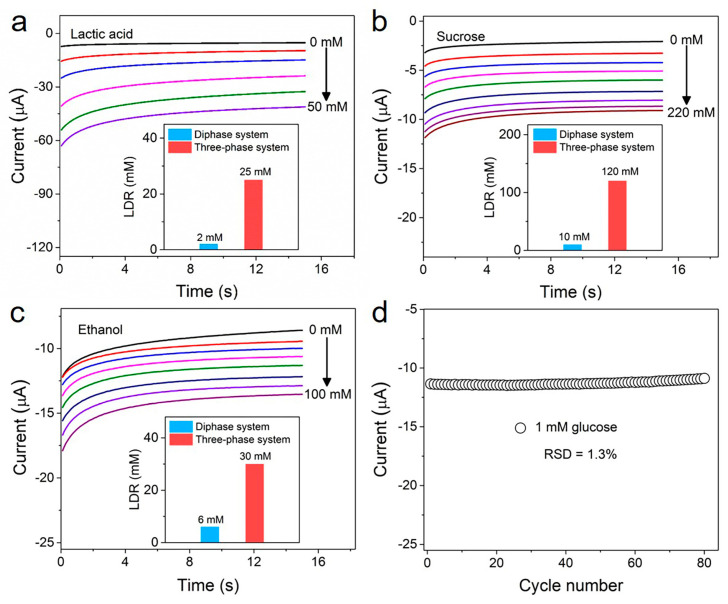
(**a**–**c**) Amperometric i-t curves of the LIG-based three-phase biosensor for lactic acid, sucrose and ethanol detection, respectively. Insets show the linear detection range (LDR) of the diphase system (blue columns) and three-phase system (red columns) corresponding to lactic acid, sucrose and ethanol, respectively. (**d**) The same LIG-based three-phase electrode was measured in 1 mM of glucose 80 successive times. The relative standard deviation is 1.3%.

The LIG-based biosensor presented here can be employed for other oxidase-based bioassays. As shown in Figure 5a–c and Figure S12, the LIG-based three-phase biosensors with corresponding oxidase layers were used to measure lactic acid, sucrose and ethanol levels. The detection performances of these three-phase enzyme electrodes show a similar trend with that of glucose: the linear detection ranges were 12.5, 12 and 5 times wider than those of corresponding diphase biosensors (the inserts of Figure 5a–c). Furthermore, we further studied the repeatability of the LIG-based three-phase biosensor. Figure 5d shows an 80-times successive measurement of 1 mM of glucose concentration using the same biosensor. A relative standard deviation of only 1.3% was observed for these measurements, suggesting good repeatability.

## 4. Conclusions

In summary, we successfully prepared a superhydrophobic LIG substrate with 3D architectures via a CO_2_ laser-fabrication process and developed electrochemical oxidase biosensors with an air–liquid–solid three-phase interface based on the LIG substrate. Different from the traditional diphase enzyme biosensor, the three-phase biosensor has a constant, air phase-dependent interfacial oxygen level. This greatly enhances and stabilizes the oxidase kinetics and enables the cathodic measurement of enzymatic product H_2_O_2_ in bioassay, thereby improving the linear detection range and accuracy of the biosensor. Laser direct-writing technology provides an efficient, low-cost and customizable approach to fabricating three-phase biosensors at a large scale.

## Data Availability

Data available on request from the authors.

## Supplementary Materials

The following supporting information can be downloaded at: https://www.mdpi.com/article/10.3390/biomimetics8010026/s1, Figure S1: Fabrication process of the LIG-based three-phase biosensor. Figure S2: The elemental mapping (EDS) of hydrophobic LIG after Pt deposition. Figure S3: UV-vis absorption spectra of oxidase enzymatic product H_2_O_2_ in 20 mM glucose solution at different reaction times based on the diphase electrode. Figure S4: (**a**) UV-vis absorption spectra of different H_2_O_2_ concentrations. (**b**) The linear relationship between the absorbance and the H_2_O_2_ concentration. Figure S5: UV–vis absorption spectra of oxidase enzymatic product H_2_O_2_ at different glucose levels based on the three-phase (**a**) and diphase (**b**) electrodes, respectively. Figure S6: (**a**–**d**) The top microscopy images of the LIG substrates fabricated at 3, 5, 6 and 7 W powers. The insets show the magnified image of LIGs. (**e**) The functional relation of H_2_O_2_ production rate with glucose concentration based on the LIG-based three-phase systems at different powers. (**f**) The dependence of the sheet resistance of LIG on laser power. Figure S7: Amperometric response of the LIG-based diphase biosensor in glucose solution with concentrations from 0 to 2 mM. Figure S8: Amperometric responses of LIG-based conventional diphase biosensors in blank PBS solution with different oxygen contents. Figure S9: (**a**) Amperometric i-t curves of the LIG-based three-phase biosensor in 0.1, 0.5 and 1 mM glucose solution with various oxygen contents. (**b**) Amperometric responses of LIG-based diphase biosensors in 1 mM glucose solution with different oxygen levels. Figure S10: Amperometric i-t response of the LIG-based three-phase biosensor at 0 V in PBS on addition of 1.0 mM of glucose, followed by 0.1 mM of methanol, ethanol, sucrose, lactic acid, galactose, xylose, mannose, citric acid and ascorbic acid. Figure S11: (**a**) Amperometric i-t curves responses of the LIG-based three-phase biosensor in glucose solution with concentrations from 0 to 1 mM, (**b**) Corresponding calibration plot derived from (**a**) at 10 s. Figure S12: (**a**–**c**) Amperometric responses of the LIG-based diphase biosensor in lactic acid, sucrose and ethanol solutions, respectively. Insets are their corresponding calibration plots derived at 10 s.
