# Supplementary material for: Laser-Induced Graphene Arrays-Based Three-Phase Interface Enzyme Electrode for Reliable Bioassays
Source: Biomimetics (Basel). 2023 Jan 8;8(1):26. doi: 10.3390/biomimetics8010026 (PMC9844491; doi:10.3390/biomimetics8010026)
Supplement: Supplementary file 1 [file biomimetics-08-00026-s001.zip › biomimetics-2053658-supplementary.pdf]

Supplementary Materials

Laser-Induced Graphene Arrays-Based Three-Phase Interface Enzyme Electrode for Reliable Bioassays

Man Zhang [1], Jun Zhang [1,*], Zhenyao Ding [1], Haili Wang [1], Lihui Huang [1] and Xinjian Feng [1,2,*]

[1] College of Chemistry, Chemical Engineering and Materials Science, Soochow University, Suzhou 215000, China
[2] Innovation Center for Chemical Science, Soochow University, Suzhou 215000, China
[*] Correspondence: jzhang2017@suda.edu.cn (J.Z.); xjfeng@suda.edu.cn (X.F.)

Figure S1

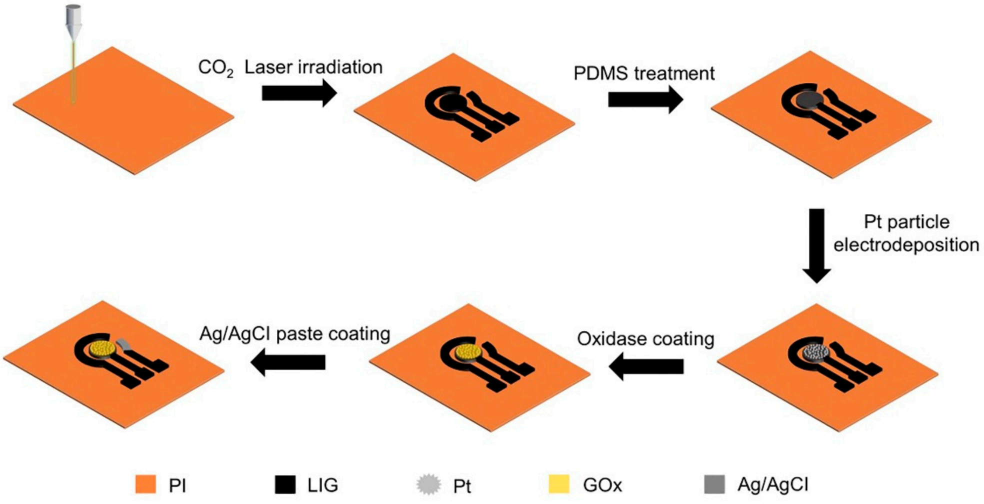

Figure S1. Fabrication process of the LIG−based three−phase biosensor.

Figure S2

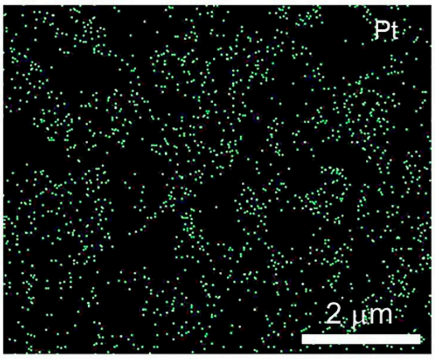

Figure S2. The elemental mapping (EDS) of hydrophobic LIG after Pt deposition.

Figure S3

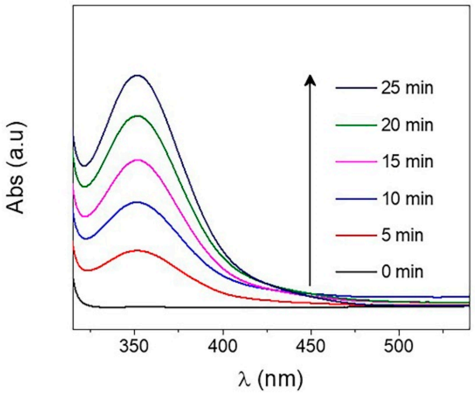

Figure S3. UV−vis absorption spectra of oxidase enzymatic product H_2O_2 in 20 mM glucose solution at different reaction times based on the diphase electrode.

Figure S4

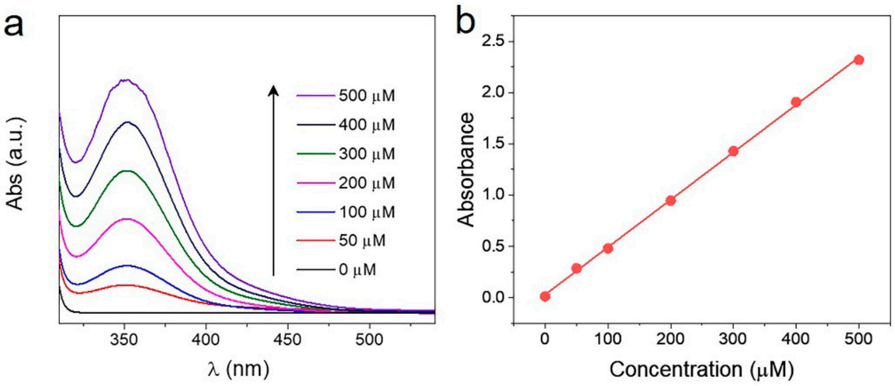

Figure S4. (a) UV−vis absorption spectra of different H_2O_2 concentrations. (b) The linear relationship between the absorbance and the H_2O_2 concentration.

Figure S5

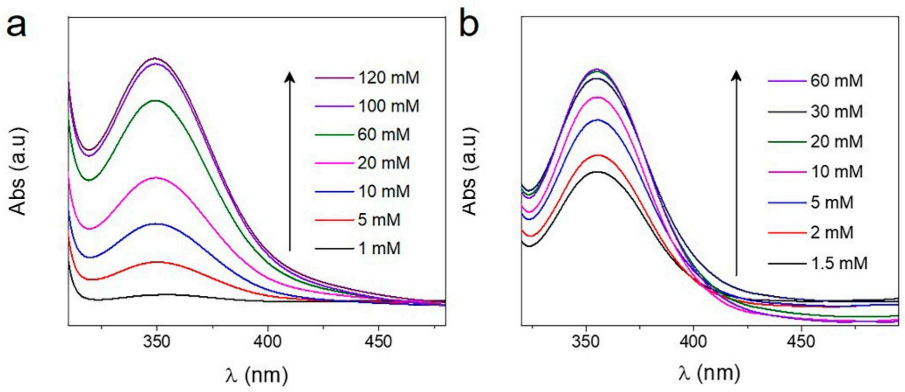

Figure S5. UV−vis absorption spectra of oxidase enzymatic product H_2O_2 at different glucose levels based on the three−phase (a) and diphase (b) electrodes, respectively.

Figure S6

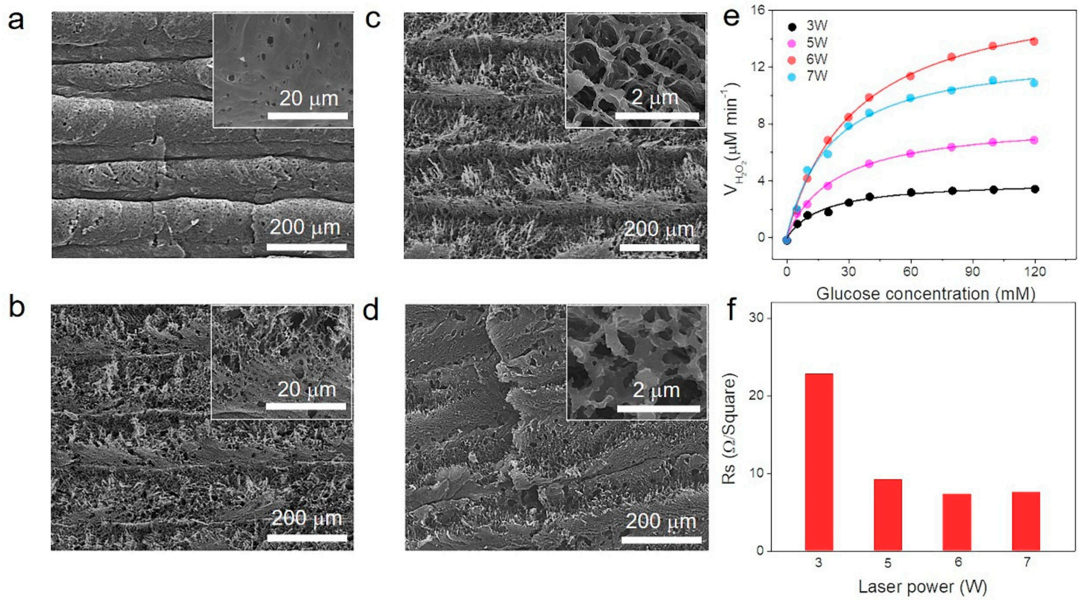

Figure S6. (a−d) The top microscopy images of the LIG substrates fabricated at 3, 5, 6, and 7 W powers. The insets show the magnified image of LIGs. (e) The functional relation of H_2O_2 production rate with glucose concentration based on the LIG−based three−phase systems at different powers. (f) The dependence of the sheet resistance of LIG on laser power.

Figure S7

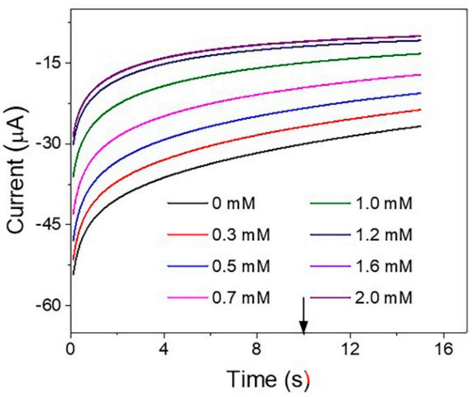

Figure S7. Amperometric response of the LIG−based diphase biosensor in glucose solution with concentrations from 0 to 2 mM.

Figure S8

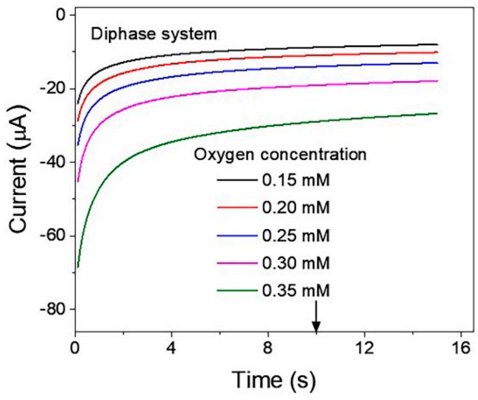

Figure S8. Amperometric responses of LIG−based conventional diphase biosensors in blank PBS solution with different oxygen contents.

Figure S9

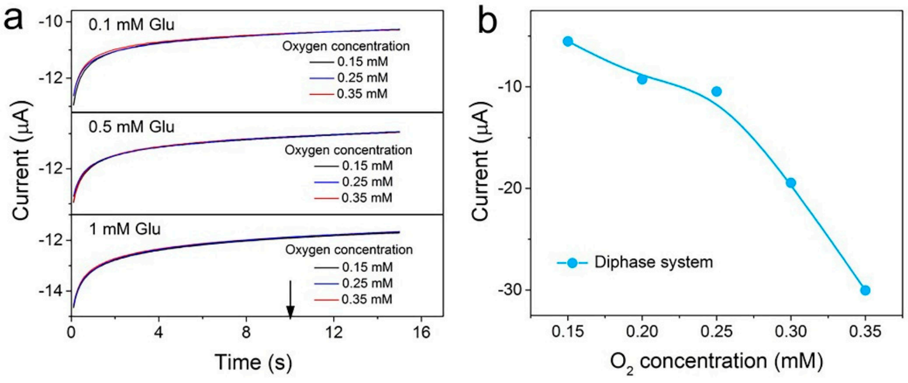

Figure S9. (a) Amperometric i−t curves of the LIG−based three−phase biosensor in 0.1, 0.5, and 1 mM glucose solution with various oxygen contents. (b) Amperometric responses of LIG−based diphase biosensors in 1 mM glucose solution with different oxygen levels.

Figure S10

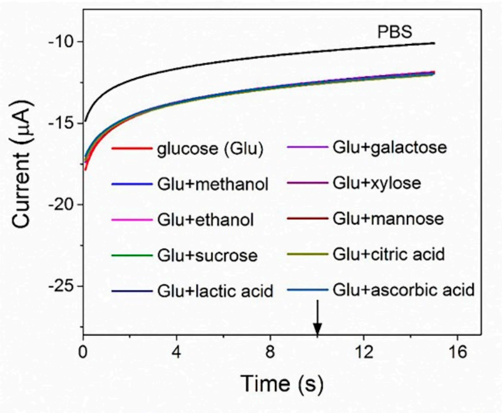

Figure S10. Amperometric i–t response of the LIG−based three−phase biosensor at 0 V in PBS on addition of 1.0 mM glucose followed by 0.1 mM methanol, ethanol, sucrose, lactic acid, galactose, xylose, mannose, citric acid and ascorbic acid.

Figure S11

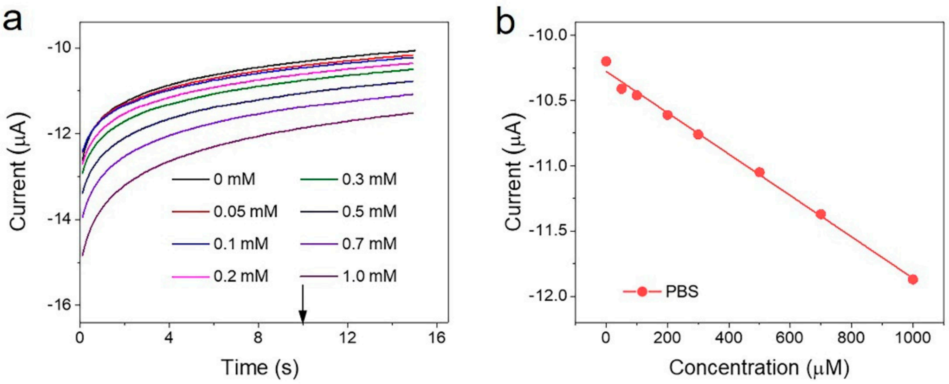

Figure S11. (a) Amperometric i−t curves responses of the LIG−based three−phase biosensor in glucose solution with concentrations from 0 to 1 mM, (b) Corresponding calibration plot derived from (a) at 10 s.

Figure S12

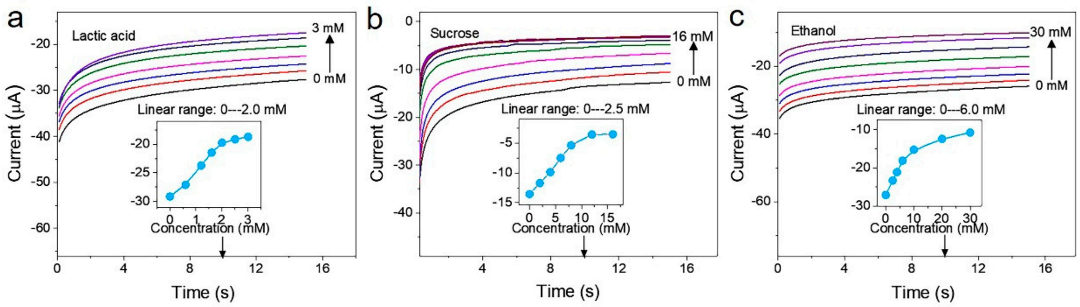

Figure S12. (a−c) Amperometric responses of the LIG−based diphase biosensor in lactic acid, sucrose, and ethanol solutions, respectively. Insets are their corresponding calibration plots derived at 10 s.
